# Plasmid Profiles of Virulent *Rhodococcus equi* Strains Isolated from Infected Foals in Poland

**DOI:** 10.1371/journal.pone.0152887

**Published:** 2016-04-13

**Authors:** Marcin Kalinowski, Zbigniew Grądzki, Łukasz Jarosz, Kiyoko Kato, Yu Hieda, Tsutomu Kakuda, Shinji Takai

**Affiliations:** 1 Department of Epizootiology and Clinic of Infectious Diseases, Faculty of Veterinary Medicine, University of Life Sciences in Lublin, Lublin, Poland; 2 School of Veterinary Medicine and Animal Science, Kitasato University, Towada, Aomori, Japan; University of Padova, Medical School, ITALY

## Abstract

*Rhodococcus equi* is an important bacterial pathogen in foals up to 6 months old, widespread in horse farms all over the world. It was found that only virulent *R*. *equi* strains expressing 15–17 kDa virulence-associated protein (VapA) and having large virulence plasmid of 85–90 kb containing *vapA* gene are pathogenic for horses. To date, 12 plasmid types have been reported in VapA positive strains from horses. There are no data concerning plasmid types of Polish field *R*. *equi* strains isolated from horses and horse farm environment. The aim of the study is to determine plasmid profiles of virulent *R*. *equi* strains isolated in Poland from dead foals as well as from soil samples taken from horse breeding farms. Plasmid profiles of 10 clinical strains derived from 8 farms and 11 environmental strains from 3 farms, confirmed as virulent by PCR, were compared with 12 reference strains containing the known plasmid size and type. Plasmid DNAs were analysed by digestion with the restriction endonucleases *Bam*HI, *Eco*RI, *Eco*T22I, and *Hin*dIII for detailed comparison and estimation of plasmid sizes. The results of RFLP analysis revealed that all except one isolates used in the study are classified as VapA 85 kb type I plasmid. One strain harboured VapA 87 kb type I plasmid. This is the first report of plasmid types of Polish field *R*. *equi* strains. The results of our preliminary investigations on horse farms located in central and eastern Poland indicate that the virulent *R*. *equi* strains thus far isolated from diseased foals and horse farms environment represent a highly uniform plasmid pattern.

## Introduction

*Rhodococcus equi* is an important bacterial pathogen in young foals up to 6 months old [1–2]. Infected animals usually demonstrate chronic and suppurative bronchopneumonia associated with high mortality rate, especially in foals not subjected to specific antibiotic therapy [3–4]. The disease causes serious economic losses in the equine breeding industry. Aside from death losses, high costs are associated with antibiotic treatment or, where implemented, preventive passive immunization strategies [2,5–6]. *R*. *equi* is widespread in horse farms all over the world including Poland [2,4,6–7]. Typically, the pathogen is found in a farm environment in the surface layer of soil contaminated with horse manure [2,4,8–9]. During hot weather dust particles coated with bacteria are inhaled by foals causing pneumonia. *R*. *equi* can be also associated with human pneumonia which is diagnosed mainly in immunocompromised patients as well as in organ transplant recipients subjected to immunosuppressive therapy [10–11]. It was found previously that only virulent *R*. *equi* strains expressing 15–17 kDa virulence-associated protein (VapA) and having large virulence plasmid of 85–90 kb containing *vapA* gene are pathogenic for horses [12]. Those VapA-positive strains are much more prevalent in farms with endemic disease comparing to farms without or with sporadic infections [13]. Both virulent and avirulent *R*. *equi* strains are taken up by alveolar macrophages in the process of phagocytosis. However, only VapA-positive bacteria are capable of blocking phagosome-lyzosome fusion leading to the disturbance of phagocyte cells and pneumonia [14–16]. Numerous studies with the use of virulent *R*. *equi* strains isolated from horses in different geographic regions in the world demonstrated the diversity of plasmid sizes as well as the prevalence of different plasmid types [9,17–19]. To date, 12 plasmid types have been reported in VapA positive strains from horses [12,19–21]. These include: 85 kb types I-IV, 87 kb types I-III and 90 kb types I-V. On the contrary, 23 plasmid types have been reported in virulent non-equine VapB-positive *R*. *equi* strains isolated from humans, swine and wild boar [22–23]. It was shown that particular plasmid types are characteristic for particular geographic regions, which can be used in epidemiological investigations [18,19]. There are no data concerning plasmid types of Polish field *R*. *equi* strains isolated from horses and horse farm environment. The aim of the study is to determine plasmid profiles of virulent *R*. *equi* strains isolated in Poland from dead foals with confirmed rhodococcosis as well as from soil samples taken from horse breeding farms.

## Materials and Methods

### Bacterial strains

Twelve reference strains of *R*. *equi* with the known plasmid size and type were used in the study. These included: ATCC 33701 (85-kb type I), 96E35 (85-kb type II), T47-2 (85-kb type III), T43 (85-kb type IV), 222 (87-kb type I), 96B6 (87-kb type II), Brazil 40 (87-kb type III), L1 (90-kb type I), S11-8 (90-kb type II), Kuma83-3 (90-kb type III), Kuma83-10 (90-kb type IV), and J21-2 (90-kb type V) [12,19–21].

### Isolation of *R*. *equi* from lung lesions of necropsied foals

**Fig 1:**
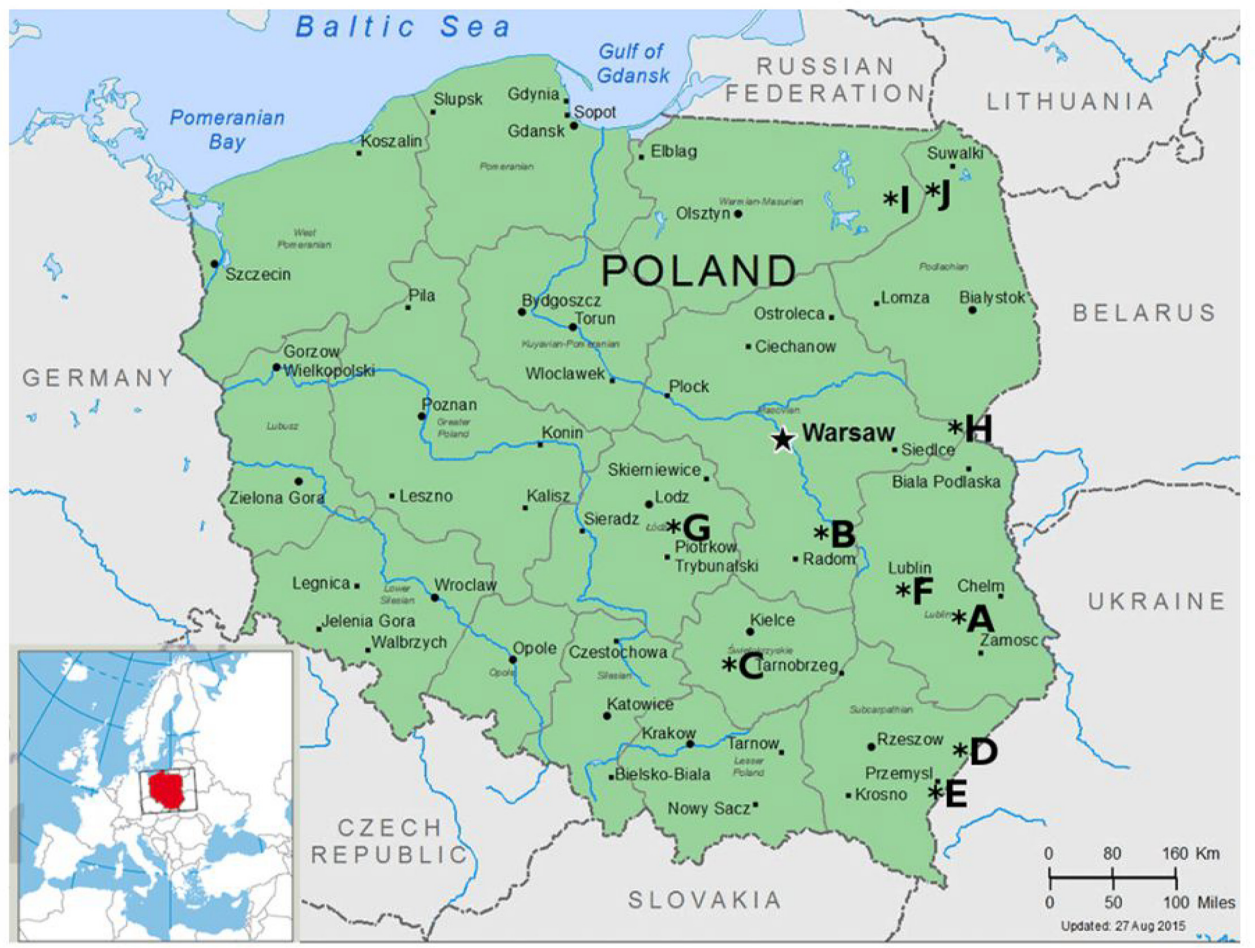
Location of studs (A-J) where clinical and environmental samples were collected.

Ten clinical isolates obtained from lung lesions of naturally infected foals during outbreaks noted in 8 Polish horse breeding farms were used (Fig 1). The details concerning isolated *R*. *equi* strains are demonstrated in Table 1. Foals were selected for inclusion in the study based on medical history provided by the farm owners, focusing on the prevalence of pneumonia in young foals. Dust pollution and insufficient stable ventilation were also taken into account as important predisposing factors. Special attention was given to the history of earlier cases of disease which were laboratory confirmed as *R*. *equi* infections. According to an agreement with the farm management, our laboratory was notified of all cases of dead foals that had presented clinical signs of pneumonia, to confirm the diagnosis. All foals were necropsied in the Clinic of Infectious Diseases, University of Life Sciences in Lublin, Poland between the years 2002–2013. Only foals presenting post-mortem changes typical for *R*. *equi* infection were enrolled in the study. Preliminary diagnosis of *R*. *equi* infection was carried out based on post-mortem examination. To collect material from lung lesions the puncture of lung abscesses was performed aseptically with the use of sterile Pasteur pipette. Each sample of pus was inoculated into 3 plates of horse blood agar. The plates were incubated at 37°C for two days. Suspicious bacterial colonies were subcultured to obtain pure culture. Identification of strains as *R*. *equi* was performed on colony morphology, Gram staining, biochemical profiling with the use of API Coryne test (BioMerieux, France, cat. no 20900) and PCR. All laboratory tests were performed with the use of reference *R*. *equi* strain ATCC 33701 as a positive control. Bacterial strains identified as virulent *R*. *equi* were stored frozen in 20% glycerol at -70°C.

**Table 1:** Plasmid profiles of *R*. *equi* isolates, clinical and environmental strains.

Number of isolate	Stud	Breed	Year of isolation	Sample origin	Age of foal	Plasmid type
Clinical samples						
1	C	Arabian	2002	lung	5 months	85kb type I
2	B	Thoroughbred	2002	lung	2 months	85kb type I
3	J	Thoroughbred/Arabian	2003	lung	3 months	85kb type I
4	C	Arabian	2003	lung	2 months	85kb type I
5	E	Thoroughbred	2004	lung	2 months	85kb type I
6	B	Thoroughbred	2004	lung	3 months	87kb type I
7	A	Arabian	2009	lung	4 months	85kb type I
8	D	Thoroughbred	2011	lung	3 months	85kb type I
9	I	Thoroughbred/Arabian	2012	lung	4 months	85kb type I
10	G	Thoroughbred	2013	lung	3 months	85kb type I
Environmental samples						
11	C	Arabian	2010	soil		85kb type I
12	C	Arabian	2011	soil		85kb type I
13	C	Arabian	2011	soil		85kb type I
14	C	Arabian	2011	soil		85kb type I
15	C	Arabian	2012	soil		85kb type I
16	A	Arabian	2013	soil		85kb type I
17	A	Arabian	2013	soil		85kb type I
18	B	Thoroughbred	2014	soil		85kb type I
19	B	Thoroughbred	2014	soil		85kb type I
20	B	Thoroughbred	2014	soil		85kb type I
21	B	Thoroughbred	2014	soil		85kb type I

The study design was reviewed and approved by the Ethics Committee of the University of Life Sciences in Lublin (Poland).

### Isolation of *R*. *equi* from soil samples

Fifty soil samples taken between the years 2010–2014 from 10 horse breeding farms (A-J) located in central and eastern Poland (Fig 1) were used for *R*. *equi* isolation. Based on medical history it was ascertained that on three of the farms (A-C) *R*. *equi* pneumonia has been enzootic for at least 20 years. On five of the farms (D,E,G,I,J) only sporadic cases of *R*. *equi* infections were noted. No clinical cases of *R*. *equi* pneumonia have been noted in other studs (F,H). Farms were selected for collection of environmental samples based on the epidemiological situation concerning previous *R*. *equi* infections in foals, as well as the environmental conditions prevailing at the studs, particularly dusty soil conducive to infection of foals, which have been raised there for many years. Five samples per stud were taken from paddocks for mares and foals and from internal roads. Soil was scraped from the upper layer of ground, up to 5 cm in depth, with the use of plastic spoon and shifted into sterile tubes. One gram of soil was serially diluted 10-fold with the use of sterile saline solution. Each dilution was inoculated into 3 plates of NANAT medium (nalidixic acid-novobiocin-actidione-potassium tellurite) as it was described by Woolcock et al. [24]. Plates were incubated at 30°C for 3 days. Bacterial colonies were counted and the number of viable organisms per gram of soil was calculated for each stud based on colony identification as *R*. *equi* (Table 2). Ten colonies per specimen were examined for *vapA* virulence marker. The multiplex PCR reaction was used to identify bacterial colonies as *R*. *equi* and to confirm the presence of *vapA* gene. Collection of soil samples and conducting of the experiment in the field were based on the permissions declared by stud owners. None of the activities undertaken during field studies involved endangered or protected species.

**Table 2:** Prevalence of virulent *R*. *equi* in soil environment of 10 studs in Poland.

Stud	Breed	Number of *R*. *equi* per gram of soil	Number of total isolates	Number (%) of virulent isolates
A	Arabian	5,5x10^3^	50	2 (4)
B	Thoroughbred	5,7x10^3^	50	4 (8)
C	Arabian	5,9x10^3^	50	5 (10)
D	Thoroughbred	4,3x10^3^	50	0
E	Thoroughbred	4,1x10^3^	50	0
F	Arabian	3,1x10^3^	50	0
G	Thoroughbred	2,8x10^3^	50	0
H	Arabian	2,9x10^3^	50	0
I	Thoroughbred/Arabian	4,5x10^3^	50	0
J	Thoroughbred/Arabian	4,2x10^3^	50	0

### Extraction of bacterial DNA

To extract bacterial DNA for PCR assay digestion of the sample with lysozyme and proteinase K using the cationic detergent CTAB (Hexadecyltrimethylammonium Bromide, Sigma, USA) was used as it was described by Sellon et al. [25]. The remaining stages of phenol-chloroform extraction and DNA precipitation were performed according to standard procedures [26]. The isolated DNA was suspended in 50 μl of sterile deionized water and stored frozen in -20°C for further analysis.

### PCR assay

Multiplex PCR reaction was used to confirm isolated strains belonging to *R*. *equi* species and to detect *vapA* gene characteristic for virulent *R*. *equi* strains. The target DNA for PCR amplification were species specific fragment of chromosomal gene coding the 16S subunit of rRNA and *vapA* gene of the virulence plasmid. The details concerning oligonucleotide primers used in the study are presented in Table 3. PCR amplification was performed with 5 μl of DNA preparation in a 50 μl reaction mixture as it was described earlier [26–27]. For each PCR reaction, the amplification of positive control sample (DNA of reference *R*. *equi* strain ATCC 33701) and negative control (with 5 μl of water instead of the DNA matrix) were performed in parallel under the same conditions. The PCR products were analysed electrophoretically in 1.5% agarose gel (Sigma) in TBE buffer in the presence of the molecular weight marker (100 bp DNA Ladder Plus, Fermentas, Lithuania). DNA fragments were visualized by UV fluorescence (Transilluminator Cole-Parmer, France) after staining with ethidium bromide (Sigma, USA).

**Table 3:** Sequences of the primers for PCR reaction.

Primer	Direction(5’→ 3’)	Sequence (5’→ 3’)	Region	Product
Rq for.	→	TCGTCCGTGAAAACTTGGG	16S rRNA	441bp
Rq rev.	←	CGACCACAAGGGGGCCGT	16S rRNA	441bp
Vp1	→	GAGGGATCCGGTTCTCGTAACGCTACAATC	vap-A	875bp
Vp2	←	TTTGAATTCTCTACACCCACCTCACACCTT	vap-A	875bp

### Isolation of plasmid DNA

Plasmid DNA was isolated from *R*. *equi* by the alkaline lysis method [28] with some modifications, as described previously [29]. The homogeneity of isolated plasmids was confirmed based on their electrophoretic patterns after separation of plasmid DNA in 0,7% agarose gel, as described previously [21].

### RFLP analysis

Plasmid DNAs were analysed by digestion with the restriction endonucleases *Bam*HI, *Eco*RI, *Eco*T22I, and *Hin*dIII for detailed comparison and estimation of plasmid sizes [20]. Samples of the plasmid preparations were separated on 0.7% or 1.0% agarose gels at approximately 5 V/cm for 2 h.

## Results

### Isolation of *R*. *equi* from lungs of dead foals

Ten clinical *R*. *equi* isolates were used in the study (Table 1). All strains have been isolated from dead foals presenting intravitally clinical signs of pneumonia. Post-mortem examination revealed changes in the lung tissue typical for *R*. *equi* infection. The pure culture of bacteria were isolated from each sample of pus taken aseptically from the lung abscesses. The results of multiplex PCR reaction confirmed all isolated bacteria as belonging to virulent, *vapA*-positive *R*. *equi* strains (Table 1).

### Isolation of *R*. *equi* from soil samples

Eleven environmental virulent *R*. *equi* strains isolated from soil samples taken from three farms (A,B,C) (Table 2) were used in the study. To collect soil samples 10 horse farms with and without previous history of *R*. *equi* infections were selected. In the preliminary studies the mean concentration of *R*. *equi* in soil was investigated by culture examination with the use of NANAT medium. The mean number of *R*. *equi* and the percentage of virulent bacteria isolated from the soil are presented in Table 2. Data presented in the table show that the concentration of *R*. *equi* in the soil taken from 10 horse farms were different in particular stud and ranged between 2,8x10^3^ cfu and 5,9x10^3^ cfu per 1.0 g of soil. Higher concentration of *R*. *equi* was found in soil samples taken from farms with a history of endemic disease in past years as compared to farms with sporadic cases or free from disease. It was found that only 3 horse farms were contaminated with virulent *R*. *equi*. Although the concentration of *R*. *equi* in the soil of endemic farms is high, as it is shown in the Table 2, the percentage of virulent organisms is low and ranges between 4–10%.

### RFLP analysis

**Fig 2:**
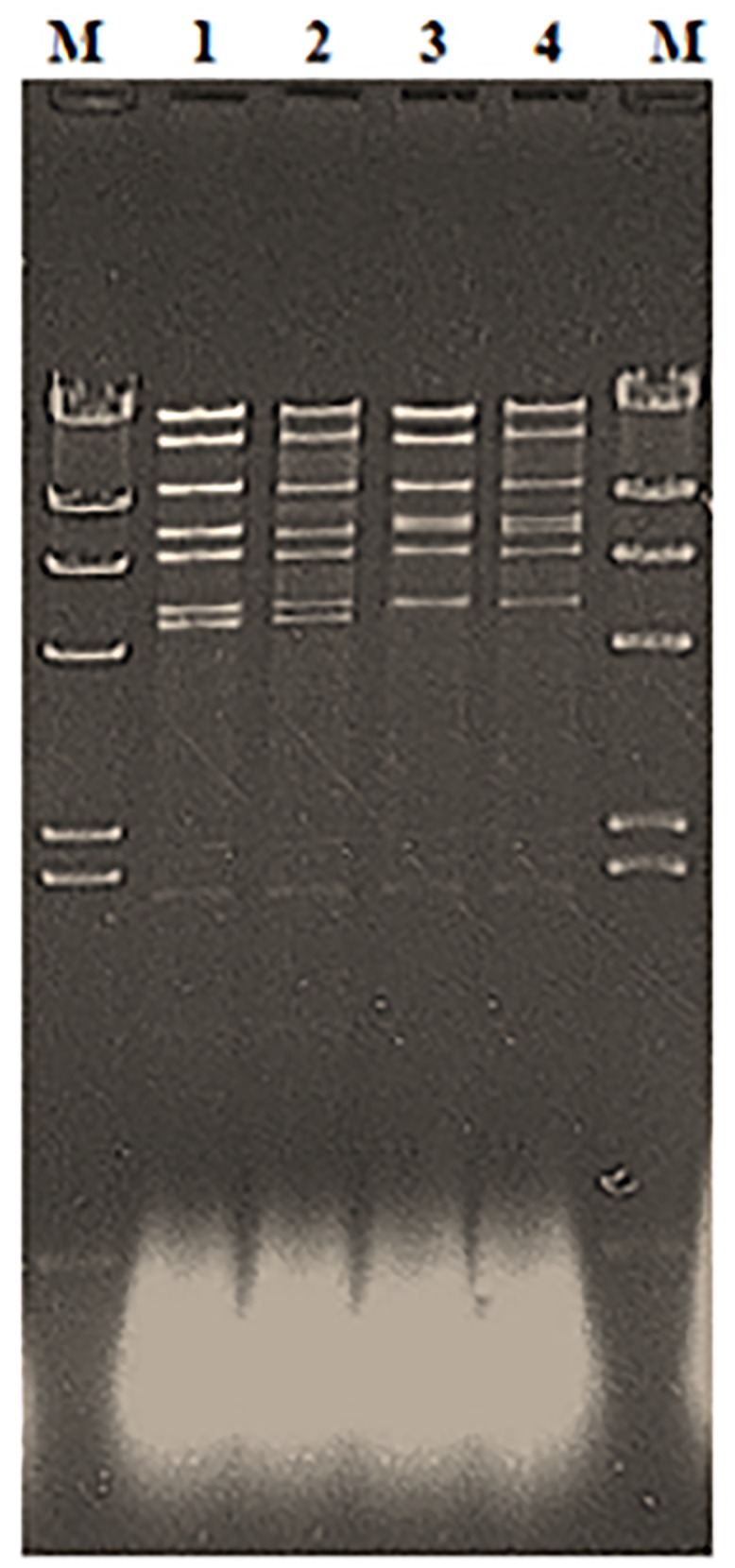
*Eco*RI restriction fragments of the plasmid types of *R*. *equi* isolates. Lane 1, strain ATCC 33701; lane 2, strain 3 (85 kb plasmid type 1); lane 3, strain 222; lane 4, strain 6 (87 kb plasmid type 1); lanes M, the markers (*Hin*dIII digestion products of bacteriophage lambda DNA).

Plasmid profiles of 10 clinical and 11 environmental *R*. *equi* isolates were investigated with the use of agarose gel electrophoresis technique. The results obtained in the study are presented in Table 1 and in Fig 2. As it is shown in the Table 1 all except one *R*. *equi* isolates harboured the same plasmid size of approximately 85 kb. This plasmid size is represented by reference *R*. *equi* strain ATCC 33701. One strain labelled as no 6 harboured larger virulence plasmid of approximately 87 kb, which corresponded in size to the reference strain 222. The results of RFLP analysis of Polish field virulent *R*. *equi* strains revealed that all except one isolates used in the study are classified as VapA 85 kb type I plasmid (Table 1). One strain harboured a VapA 87 kb type I plasmid. Our RFLP results show that type I of the VapA plasmid is predominant in Polish field *R*. *equi* strains and has been detected in all isolates irrespective of the plasmid size. The results of the study have also shown that the plasmid type is independent from the origin of sample taken for *R*. *equi* isolation. The same results were obtained with strains isolated from lung samples as well as from environmental samples. The same concerns the relationship between the plasmid type and date of *R*. *equi* isolation (Table 1).

## Discussion

This is the first study undertaken in Poland aiming at the characterisation of plasmid profiles of Polish field *R*. *equi* strains isolated of foals and soil samples. The investigation was based on *R*. *equi* strains isolated from 8 horse farms located in the central and eastern part of Poland (Table 1, Fig 1). Our preliminary results indicate that virulent *R*. *equi* strains isolated from diseased foals and horse farms environment represent a similar plasmid pattern, because all isolates except one isolated so far in Poland were classified as belonging to VapA 85 kb type I plasmid. In contrast, we detected one virulent strain belonging to VapA 87 kb type I plasmid. This result suggest possible contribution of French or Italian isolates as a cause of *R*. *equi* infections in Polish horse farms. In fact, the horse farm on which this specific plasmid type was confirmed has been largely used as a pension for horses, including pregnant mares. The animals introduced to the pension are usually the property of private owners, and their origin is difficult to establish. It is likely that adult horses originating in regions where the VapA 87 kb type I plasmid is prevalent could be carriers of virulent *R*. *equi* and a source of contamination of the environment. However, we did not detect the same plasmid type in soil samples taken from this stud. Hence it can be assumed that the introduction of this plasmid type into the stud was probably incidental and did not result in long-lasting prevalence of the bacteria in the farm environment. According to Makrai et al. [22] and Duquesne et al. [18] this plasmid type is widespread throughout the world and it is the most prevalent in *R*. *equi* strains isolated of foals from France, Italy, Turkey, both American continents and Australia.

To date, at least 12 different plasmid profiles have been detected in virulent *R*. *equi* strains worldwide [12,19–21]. The similarities of *R*. *equi* strains isolated in Poland to these originated from other European countries may indicate their common origin and ancestry.

In the previous similar studies the geographic differences in the distribution of virulence associated plasmids all over the world have been clearly shown [9,12,18–21,30–31]. These studies indicate that clinical *R*. *equi* isolates from American continents, Australia and Europe belong mainly to the 85 kb type I and 87 kb type I plasmid types. However, an 85 kb type II plasmid has been found so far only in France. The similar plasmid types, classified as 85 kb type III and IV were found in *R*. *equi* strains from North America [19]. On the contrary, different plasmid types are characteristic for *R*. *equi* strains isolated in Japan and Korea [21]. Both clinical and environmental isolates from these countries belong to 87 kb type II or 90 kb types I-IV. However, an 85 kb plasmids typically found in Europe have never been isolated in Japan [19]. This result may indicate that both groups of *R*. *equi* strains represent different genetic lineages and specific geographic location of Japan can be responsible for restriction in the prevalence of particular type strains in this country. Results of studies undertaken with strains isolated in Japan and Korea suggest their common origin [21]. According to Takai et al. [32] one of the possible explanation may be a historical introduction of both type strains into these countries through Mongolian horses. However, there are no data at the moment about plasmid profiles of *R*. *equi* strains from Mongolia and China, which could to confirm this evidence.

Our study has several limitations. First, the examination was based on clinical and environmental samples taken from relatively small number of horse farms located in the central and eastern part of Poland. There are several reasons for this restriction. First, the epidemiological situation concerning the prevalence of *R*. *equi* infections on Polish horse farms is poorly understood. Also, despite the enzootic status of some studs, generally only isolated clinical cases of *R*. *equi* infection are noted. Additionally, on many farms suspected cases of pneumonia in foals are treated with antibiotics and are not reported. In the case of environmental samples, the main limitation is the common prevalence of avirulent *R*. *equi* strains in the soil. Therefore selection of VapA-positive bacteria may be difficult in culture examination. Another limitation concerns statistical analysis of results, which may be lacking due to the small number of *R*. *equi* strains isolated from clinical cases in foals and from breeding farm environments. Further long-term studies, including more virulent *R*. *equi* strains representing the majority of Polish horse farms, are necessary to assess real distribution of different plasmid types within *R*. *equi* strains in Poland.

In summary, the analysis of plasmid profiles of *R*. *equi* strains isolated in Poland from necropsied foals and from horse breeding environment revealed the prevalence of two plasmid types, VapA 85 kb type I and VapA 87 kb type I. Taking into account the results of previous investigations undertaken with *R*. *equi* strains isolated in different continents we can conclude that Polish field *R*. *equi* strains do not represent any new plasmid type, not found earlier elsewhere. The characteristics of virulence plasmids of *R*. *equi* strains isolated in different parts of the world is fundamental in epidemiological tracing of the source of infection for horses, as well as for other animal species and humans.
